# The ApoA-I mimetic peptide 4F attenuates in vitro replication of SARS-CoV-2, associated apoptosis, oxidative stress and inflammation in epithelial cells

**DOI:** 10.1080/21505594.2021.1964329

**Published:** 2021-09-08

**Authors:** Kelesidis Theodoros, Madhav Sharma, Petcherski Anton, Cristelle Hugo, O’Connor Ellen, Nan W Hultgren, Eleni Ritou, David S Williams, Shirihai Orian S, Reddy Srinivasa T

**Affiliations:** a Department of Medicine, David Geffen School of Medicine, University of California Los Angeles, Los Angeles, California, USA; b Molecular Toxicology Interdepartmental Degree Program, University of California Los Angeles, United States; c Department of Ophthalmology, David Geffen School of Medicine, University of California Los Angeles, Los Angeles, California, USA; d Department of Molecular and Medical Pharmacology, David Geffen School of Medicine, University of California, Los Angeles, California, USA

**Keywords:** COVID-19, SARS-CoV-2, ApoA-I mimetic peptides, 4F, therapeutics, inflammation, apoptosis, antivirals, antioxidants

## Abstract

An oral antiviral against SARS-CoV-2 that also attenuates inflammatory instigators of severe COVID-19 is not available to date. Herein, we show that the apoA-I mimetic peptide 4 F inhibits Spike mediated viral entry and has antiviral activity against SARS-CoV-2 in human lung epithelial Calu3 and Vero-E6 cells. In SARS-CoV-2 infected Calu3 cells, 4 F upregulated inducers of the interferon pathway such as MX-1 and Heme oxygenase 1 (HO-1) and downregulated mitochondrial reactive oxygen species (mito-ROS) and CD147, a host protein that mediates viral entry. 4 F also reduced associated cellular apoptosis and secretion of IL-6 in both SARS-CoV-2 infected Vero-E6 and Calu3 cells. Thus, 4 F attenuates *in vitro* SARS-CoV-2 replication, associated apoptosis in epithelial cells and secretion of IL-6, a major cytokine related to COVID-19 morbidity. Given established safety of 4 F in humans, clinical studies are warranted to establish 4 F as therapy for COVID-19.

## GRAPHICAL ABSTRACT

**Figure:**
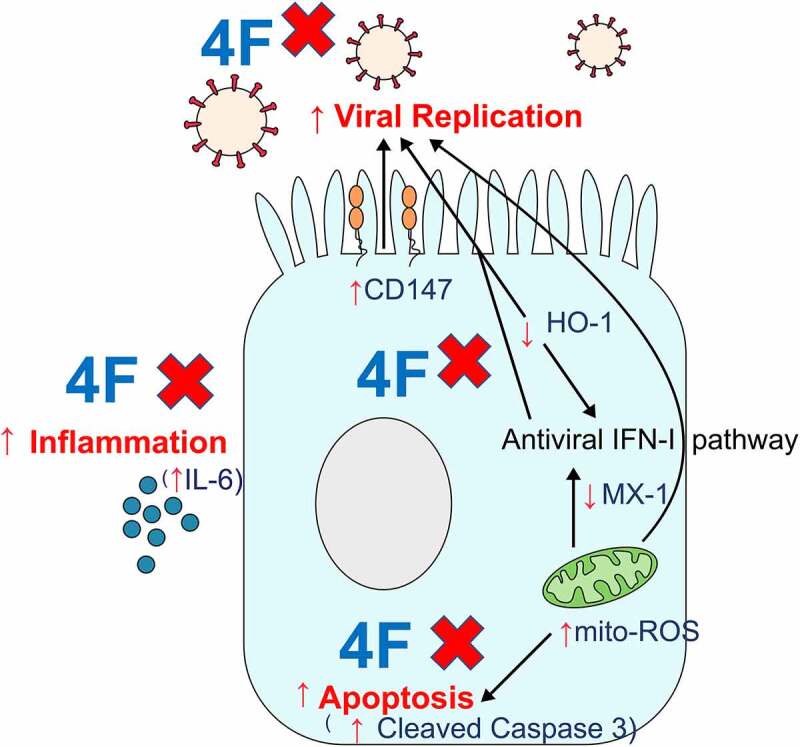

## Introduction

COVID-19 is more lethal in older patients with cardiovascular disease (CVD) and comorbidities[1] but there is no therapy that has favorable therapeutic effect on both comorbidities and COVID-19. Vaccines may have suboptimal efficacy against SARS-CoV-2 variants and in immunocompromised patients and may not be accepted uniformly by all people. Current antivirals against SARS-CoV-2 have major limitations. Hydroxychloroquine does not have efficacy against COVID-19 in clinical trials. Remdesivir needs to be administered intravenously, and studies have shown controversial efficacy [2,3]. Oral JAK inhibitors like baricitinib have off-target effects in multiple organ systems and can have serious side effects [4,5]. Ivermectin does not achieve any therapeutic levels in the respiratory mucosa and is associated with serious toxicity [6]. Thus, there is an unmet need to develop an efficacious, orally available, safe, novel therapeutic strategy to protect from development of COVID-19 while having favorable impact on comorbidities.

Apolipoprotein A-I (apoA-I) mimetic peptides like 4 F mimic the secondary structure of apoA-I, the major protein of high-density lipoproteins, and interfere with membrane fusion and entry of viruses into the host cell [7]. 4 F spontaneously forms an amphipathic helix whose hydrophobic domain binds tightly to and disrupts lipid membranes. 4 F binds bioactive lipids with higher affinity than full length apoA-I and has emerged as a new class of therapeutic molecules for treating inflammatory diseases including cancer, cardiovascular and inflammatory bowel disease in mice [8–12]. 4 F can attenuate gut inflammation in mice when given orally [12] and has been shown to be safe in humans in clinical trials when given orally [13] or parenterally [14]. 4 F treatment had antiviral activity *in vivo* against influenza and reduced influenza-induced lung inflammation [15]. These antiviral properties of 4 F prompted us to investigate whether 4 F has antiviral activity against SARS-CoV-2.

## Methods

### Reagents

The USA-WA1/2020 SARS-CoV-2 isolate was acquired from the Biodefense and Emerging Infections Resources of the National Institute of Health. Vero-E6 (CRL1586) and Calu3 (HTB-55) cell lines were commercially available from the American Type Culture Collection. 4 F was prepared as previously described [16,17] and was then used at 1–100 μM as described previously [16,17]. Remdesivir and DMSO were purchased from Sigma Aldrich. Remdesivir was dissolved in DMSO and was then used at 1 μM as previously described [18]. The codon optimized Spike protein plasmid HDM-SARS2-Spike-delta21 for pseudotyping was a gift from Jesse Bloom (Addgene plasmid # 155,130) [19]. The lentiviral construct pLKO-RFP-shCntrl for packaging was a gift from William Kaelin (Addgene plasmid # 69,040).

### Cell cultures

Calu3 cells and Vero E6 cells were maintained at 37°C and 5% CO_2_ in Modified Eagle Medium (MEM, Corning) supplemented with 10% Fetal Bovine Serum (FBS), penicillin (100 units/ml), and streptomycin (100 µg/ml).

### SARS-CoV-2 infection and drug treatments

All studies involving live virus were conducted after appropriate institutional biosafety approvals. TCID_50_ assay was used to determine virus titer. Cell cultures were infected with SARS-CoV-2 viral inoculum (MOI of 0.1; 100 µl/well) in 96 well plates. Media alone was used for mock infection. 48 hours before infection, Vero E6 cells were plated at 20,000 cells/well and Calu3 were plated at 50,000 cells/well in a 96-well plate. Medium with a concentration range of 4 F versus vehicle (deionized water) controls were added 24 hours before infection. After three washes, cells were then inoculated with MOI 0.1 PFU/cell of SARS-CoV-2 at 37°C in serum free medium for one hour. Plates were rocked every 15 min and after 60 min, virus was removed, cells were washed with Phosphate buffered saline (PBS) once to remove unbound virus. Medium containing vehicle control (dH2O), 4 F or remdesivir was added onto the cells, and cells were incubated for 24–48 hours at 37°C. Drug effects were measured relative to vehicle control. To simulate the use of 4 F as antiviral agent for preexposure prophylaxis, cells were treated with 4 F triplicate per dilution for 24 hours before infection. At 48 hours post infection cells and cell culture supernatants were harvested for downstream applications.

### Preparation of SARS-CoV2 Spike protein pseudotyped lentivirus

To generate Spike protein pseudotyped lentivirus, 293 T cells were transfected with pLKO-RFP-shCntrl, HDM-SARS2-Spike-delta21, and pPAX2 plasmids with Lipofectamine 3000 (ThermoFisher) overnight as previously described [19]. Viral supernatants were collected at 48- and 72-hour post-transfection and combined. The viral supernatant was concentrated via ultracentrifugation at 25,000 RPM for 90 minutes at 4°C, reconstituted in 1X DPBS and stored at −80°C until use. For titer, Vero-E6 cells were seeded in 96-well plates at 2,500 cells/well 24 hour prior to transduction. Spike protein pseudotyped lentivirus was added at 1:10, 1:50 and 1:250 dilution and incubated for 24 hours before recovery. Cell nuclei were stained with 1 μg/mL Hoechst 33,342 for 30 min. Hoechst and RFP fluorescence signal were imaged with a Perkin-Elmer Operetta high-content imager and the percentage of RFP-positive cells quantified using CellProfiler 2.0 as previously described [20].

### SARS-CoV2 spike protein pseudotyped lentivirus entry experiments in Vero-E6 cells

Vero-E6 cells cells were plated at a density of 2500–5,000 cells/well into Costar black-walled imaging bottom 96-well plates and allowed to attach over-night. Medium with a concentration range of 4 F (1–10 μM) versus vehicle (deionized water) controls were added 24 hours before transduction. Pre-treatment media was removed and SARS-CoV2 Spike Protein pseudotyped lentivirus (MOI 0.5–1) was added in the presence of 4 F containing or dH_2_O containing media as control for 1–4 hours. After one PBS wash, media containing 4 F or dH_2_O was reintroduced for 24 h before cells were imaged for immunofluorescence or were processed for flow cytometry as described above.

### Cell cytotoxicity assay

Cells were plated and treated with drugs as described above and after 24 hours, cell viability was measured via the XTT Cell Proliferation Assay Kit (ATCC® 30–1011 K™) on a Synergy 2 Biotek microplate reader (Biotek Inc) following the manufacturer’s protocol.

### RNA extraction and quantitative polymerase chain reaction (q-RT-PCR)

Total RNA was extracted from cells in TRIzol (Invitrogen) using the Direct-zol RNA Miniprep kit (Zymo Research) or the RNeasy Mini Kit (Qiagen 74,104) according to the manufacturer’s instructions. RNA was reverse transcribed into cDNA using oligo d(T) primers using Superscript II Reverse Transcriptase (Thermo Fisher). Quantitative real-time PCR was performed using Green qPCR Master Mix (Thermo Fisher) and primers specific for SARS-CoV-2 as well as GADPH transcripts. The following primers were used: h-GAPDH-F: CCACCTTTGACGCTGGG; h-GAPDH-R: CATACCAGGAAATGAGCTTGACA; 2019-nCoV_N1-F: GACCCCAAAATCAGCGAAAT, 2019-nCoV_N1-R: TCTGGTTACTGCCAGTTGAATCTG. All qRT-PCR reactions were performed using BIO-RAD CFX96 Touch Real-Time PCR Detection System on 96-well plates. Each 20 μL reaction mixture contained 0.4 μL each of 10 μmol/L forward and reverse primer, 4.2 μL of RNase-free water, 5 μL of cDNA as template and 10 μL of 2X SYBR Green RT-PCR Master Mix (Thermo). The reactions were incubated at 45°C for 10 min for reverse transcription, 95°C for 2 min, followed by 40 cycles of 95°C for 15 s and 60°C for 60 s. Gene expression fold change was calculated with the Delta-delta-cycle threshold (DDCt) method. Viral RNA levels were normalized to GADPH as an endogenous control and depicted as fold change over mock infected samples. Error bars indicate the standard error of means from three biological replicates.

### Imaging and immunofluorescence

Cells were fixed with 4% paraformaldehyde in PBS for 20 minutes and were then permeabilized and blocked for 1 hour with 0.3% Triton X-100, 2% bovine serum albumin, 5% donkey serum and 5% goat serum. The Spike S primary antibody (1:100, Sino Biologicals Cat# 40,150-R007) was added to samples overnight at 4°C. Samples were rinsed 5 times for 2 minutes each with PBS containing 0.3% Triton X-100. The goat anti-rabbit Alexa Fluor® 488 IgG (Abcam, Cat# ab150077) secondary antibody was diluted 1:1000 in blocking buffer and was then added for 2 hours at room temperature. Samples were then rinsed 5 times for 2 minutes each with PBS containing 0.3% Triton X-100, followed by DAPI diluted in PBS at 1:5000 for 10 minutes. Images were obtained using Zeiss LSM880 confocal microscope using the Airyscan detector and a 20X air objective (Carl Zeiss GmbH, Jena, Germany) and were quantified using CellProfiler 2.0. Alternatively, plates were imaged using the Operetta (PerkinElmer, Waltham, MA) system.

### Mitochondrial reactive oxygen species analysis

Cells were stained with 5 μM MitoSOX Red Mitochondrial reactive oxygen species (mito-ROS) indicator for 30 min at 37°C at 48 hours post infection. Cells were then washed with PBS, fixed with 4% paraformaldehyde for 30 min at 4°C and transferred to FACS tubes. Cells were then analyzed using an LSR Fortessa flow cytometer and FACSDiva software (Becton & Dickinson, San Diego, CA, USA), and data were analyzed using FlowJo software.

### Flow cytometry

SARS-CoV-2 infected cells were fixed with 4% paraformaldehyde for 20 min at 4°C within the UCLA BSL3 high-containment facility and were transferred in polypropylene E-tubes to BSL2 containment facility for further processing. The single cell suspensions were incubated with viability dye (Fixable Ghost Dye™ Red 780, Tonbo Biosciences) for 20 min in the dark at room temperature. The following antibodies were added to each tube and incubated in the dark for 20 minutes on ice: PE/Cyanine7 mouse anti-human CD147 antibody (clone HIM6 from Biolegend, San Diego, CA), mouse anti-human HO-1 antibody (clone HO-1-2 from Enzo Life Sciences) conjugated with Mix-n-Stain CF647 Antibody Labeling Kit from Biotium Inc (Hayward, CA), rabbit anti-human MX1 polyclonal antibody (from Proteintech, 13,750-1-AP), rabbit anti-SARS-CoV-2 (2019-nCoV) Spike S1 Antibody (Clone R007, Cat# 40,150-R007 from Sino Biologicals conjugated with Mix-n-Stain CF488 Antibody Labeling Kit from Biotium Inc (Hayward, CA), mouse anti-SARS-CoV/SARS-CoV-2 spike S antibody (clone 1A9 from GeneTex), Alexa Fluor® 647 Cleaved Caspase-3 (Asp175) (Clone D3E9 from Cell Signaling Technology, Cat# 9602S), Alexa Fluor® 647 Endorepellin/Perlecan/Heparan Sulfate Proteoglycan Antibody (clone A7L6 cat# NBP2-44,448). Secondary antibodies were goat anti-rabbit Alexa Fluor® 488 IgG (Abcam, Cat# ab150077), goat anti-mouse Alexa Fluor® 546 (Thermo Fisher Scientific Cat# A11003) and goat anti-rabbit DyLight® 650 (Abcam, Cat# ab96902). After 20 min, the cells were washed twice with PBS, were suspended in 200 μL of ice-cold PBS buffer and were transferred to fresh tubes for FACS analysis. Samples were acquired using an LSR Fortessa flow cytometer and FACSDiva software (BD Biosciences). Data were analyzed using FlowJo software. At least 10,000 cells were acquired for each analysis, and each representative flow plot was repeated more than 3 times. Single stain and also fluorescence minus one (FMO) control were used to determine positive staining for a protein. The difference in fluorescence intensity compared to the negative control (DMFI or % positive cells for each protein of parent cell population) was reported for each sample.

### Biomarkers of inflammation

Protein levels of secreted IL-6 were determined in cell culture supernatants using ELISA kits according to the manufacturer (R&D). Protein levels of cytokines (IL-1 beta, IL-8, TNF-alpha, IL-10) in cell culture supernatants were determined using the human magnetic Luminex performance assay kits (LXSAHM) according to the manufacturer instructions (R&D).

### Statistics

Unless noted, error bars in all figures represent mean and standard error of means (SEM). In the figures, p-values are presented for comparisons between treatment groups and controls and are denoted by asterisks. Each experiment contains at least three biological replicates (number of wells) and each analysis contains at least three independent experiments. For analysis of data that contains more than 2 groups, the Kruskal-Wallis test was performed to compare samples; if these comparisons had a *p* value less than 0.05 then Mann-Whitney *U* tests were used to compare statistical difference between 2 groups. *p* values less than 0.05 by Kruskal-Wallis or Mann-Whitney were considered significant. In the setting of exploratory approach we did not adjust for multiple comparisons since commonly-used multiple testing adjustment methods assume independence of tests, which in protein expression studies and in our explored pathways translates to a questionable assumption that all explored measures operate independently [21]. Instead, consistency between 2 independent experiments, direction, and magnitude of the correlation coefficient in conjunction with the nominal p values were considered in order to help distinguish true and false-positive findings. All analyses were performed with Graphpad Prism version 8.0.

## Results

### 4 F has antiviral activity against SARS-CoV-2 in epithelial cells

**Figure 1.**
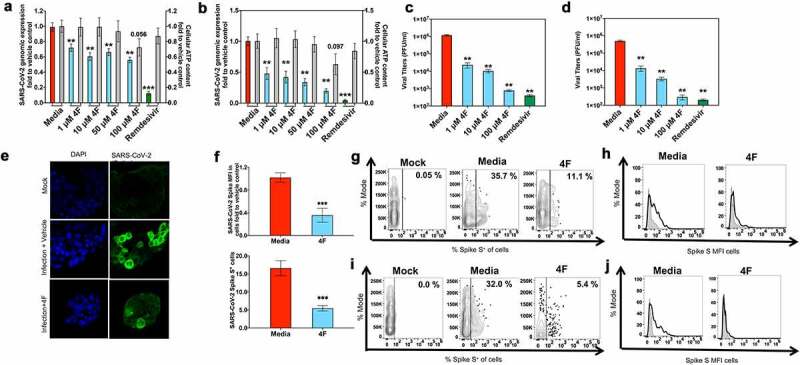
4 F restricts SARS-CoV-2 infection in epithelial cells. Vero E6 and Calu3 cells were infected with SARS-CoV-2 (MOI 0.1) and treated with media alone (vehicle), remdesivir or 4 F (1–100 μM) as in methods. qRT-PCR analysis of Vero E6 (a) and Calu3 (b) cells 48 hours post infection. The graph depicts the relative amount of SARS-CoV-2 Spike S normalized to human GADPH. Relative viral quantification was done compare to the positive control (infected cells treated with vehicle). Viral titers were determined in supernatants at 48 hours post infection in Vero E6 cells (c) and Calu3 cells (d). **E, F**. Confluent Calu3 cells treated with vehicle (media) or 4 F (10 μM) were fixed 48 h post-infection (hpi) followed by processing for immunostaining with the SARS-CoV-2 Spike S antibody and DAPI. The percentage of SARS-CoV-2-infected cells and the median fluorescence intensity of SARS-CoV-2 Spike S protein per cell (MFI in arbitrary units) were determined. **E**. Representative images of the indicated conditions. Scale bar: 200 µm. **F**. Summary immunofluorescence data (% of SARS-CoV-2^+^ cells and SARS-CoV-2 Spike S MFI in cells fold to vehicle control (media). **G-J**. Flow cytometry was used in Vero E6 cells (g, h) and Calu3 cells (i, j) to determine the percent of Spike S protein^+^ viable cells and the MFI of Spike S protein that were compared to a negative cell population (fluorescence minus one negative control for staining shown in light gray). Representative data are shown for Vero E6 (**G, H**) and Calu3 (**I, J**) uninfected (mock) and SARS-CoV-2^+^ infected cells treated with media versus 4 F (10 μM). Data represent the mean ± SEM, representing three independent experiments performed in at least two technical replicates. The Kruskal-Wallis statistical test was used to compare 3 groups and the Mann-Whitney was used to compare each group relative to the vehicle control and the p value for this comparison is shown above each column (*p < 0.05, **p < 0.01, ***p < 0.001)

Vero E6 and human lung epithelial Calu3 cells were pretreated for 24 hours (h) with 4 F (1–100 μM) as previously [17] and were subsequently infected with a clinical isolate of SARS-CoV-2 [multiplicity of infection (MOI) of 0.1] for 48 h in 4 F- or remdesivir- containing medium. Remdesivir was included as a positive control for antiviral effect [18]. Using qPCR that assessed viral genomic RNA in cell lysates we showed that 4 F inhibited SARS-CoV-2 in both Vero E6 (Figure 1a) and Calu3 cells (Figure 1b). There was no observed cytotoxicity associated with 4 F in similarly treated uninfected cultures across the 1–10 μM dose range (Figure 1a, b). These results were corroborated by a reduction in viral titer, where 4 F at concentrations 1–100 μM displayed similar reduction in viral titer in both Vero E6 (Figure 1c) and Calu3 cells (Figure 1d). A concentration 10 μM was chosen for all future experiments. Given that levels of viral proteins may better reflect cellular coronavirus infection than viral genomic RNA [22], using immunofluorescence, we confirmed that 4 F had antiviral activity in Calu3 cells (Figure 1e, f). Given that immunofluorescence is semiquantitative method for assessment of protein levels, we used flow cytometry to characterize the cellular protein levels of the Spike S protein in infected cells. We confirmed immunofluorescence data (Figure 1f) at the single cell level in viable cells, that 4 F induced a more prominent reduction in the percent of infected cells that were positive for Spike S protein compared to the Spike S MFI in infected Vero E6 (Figure 1g, h) and Calu3 (Figure 1i, j) cells. Our data are consistent with prior evidence that apoA-I mimetic peptides have antiviral activity against viruses [7,15].

### Impact of 4 F on viral entry of SARS-CoV-2 infected epithelial cells

**Figure 2.**
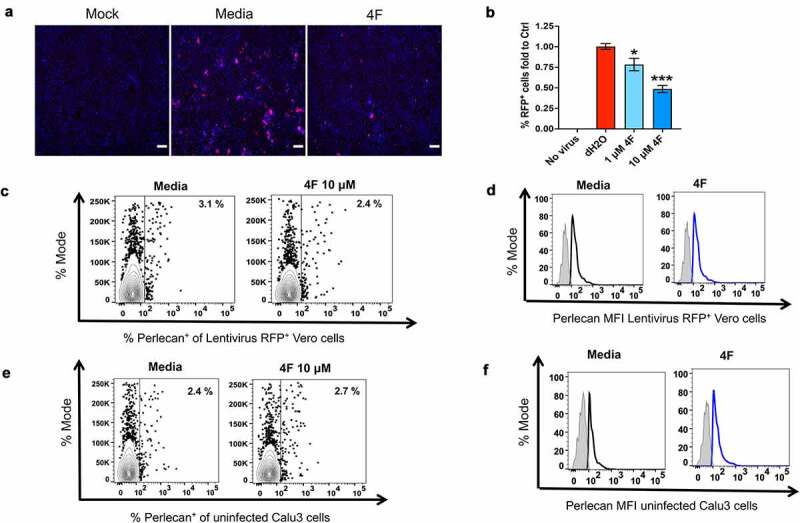
Impact of 4 F on SARS-CoV-2 Spike protein entry and host heparan sulfate proteoglycans in epithelial cells. A, B. Confluent Vero E6 cells were transduced with RFP expressing SARS-CoV-2 spike-bearing pseudotyped lentivirus and treated with media alone (vehicle), or 4 F as in methods. Vero E6 cells treated with vehicle (media) or 4 F (1 μM and 10 μM) were fixed 24 h post-infection (hpi) followed by assessment of the fluorescent (RFP) SARS-CoV-2 Spike S protein by fluorescence microscopy (a, b). **A**. Representative fluorescence images of Vero cells treated as shown. RFP fluorescence is shown in red and nuclei were stained with Hoechst (Blue). Scale bar: 100 µm. **B**. Summary fluorescence data (% of RFP^+^ cells) fold to vehicle control (media) from two independent experiments. Data represent the mean ± SEM, representing two independent experiments performed in at least three technical replicates. Kruskal-Wallis statistical test was used to compare 3 groups and the Mann-Whitney was used to compare each group relative to the vehicle control and the p value for this comparison is shown above each column (*p < 0.05, **p < 0.01, ***p < 0.001). **C, D**. Perlecan is a major host heparan sulfate proteoglycans (known mediators of SARS-CoV-2 entry) found within all cell surfaces. Flow cytometry was used in Vero E6 cells transduced with SARS-CoV-2 spike-bearing pseudotyped lentivirus and treated with media alone (vehicle), or 4 F (1, 10 μM) to determine the percent of perlecan^+^ cells in RFP^+^ cells (c) and the MFI of perlecan in RFP^+^ cells compared to a negative cell population (fluorescence minus one negative control for staining shown in light gray) (MFI)(d). Representative data from 3 independent experiments are shown. **E, F**. Flow cytometry was used in uninfected Calu3 cells treated with media alone (vehicle), or 4 F (10 μM) to determine the percent of perlecan^+^ cells (e) and the MFI of perlecan (f) compared to a negative cell population shown in light gray) (MFI). Representative data from 3 independent experiments are shown

Given that 4 F induced a more prominent reduction in the percent of infected cells that were positive for Spike S protein compared to the Spike S MFI in infected epithelial cells and that 4 F can impact cellular membranes [23] that are important for entry of pathogens, we studied the impact of 4 F on viral entry in epithelial cells. To gain further insight into the impact of 4 F specifically on SARS-CoV-2 Spike protein-mediated viral entry and not cell-to-cell spread and productive infection, we utilized SARS-CoV-2 Spike pseudotyped lentiviral particles expressing red fluorescent protein (RFP) as a reporter gene. Pseudotyped lentivirus was added to Vero cells in the presence of 4 F to study the impact of 4 F on SARS-CoV-2 viral entry. Using fluorescence microscopy, and similarly to previous data [24], the mean transduction efficiency of the pseudotyped lentivirus (% of cells positive for RFP expression) as a measure of viral entry in epithelial cells, was 10.2% (Figure 2a, b). 4 F reduced viral entry of the pseudotyped lentivirus by a mean 22%-fold (1 μM) and a mean 52%-fold (10 μM) in a dose-dependent way (1 μM versus 10 μM) compared to the vehicle-treated infected Vero cells (Figure 2a, b). Thus, consistent with the role of 4 F on altering cellular membranes that mediate viral entry [23], 4 F inhibited *in vitro* SARS-CoV-2 Spike protein mediated viral entry in Vero cells.

### Impact of 4 F on viral attachment to the host heparan sulfate proteoglycans of uninfected and infected epithelial cells

Antimicrobial peptides or proteins such as lactoferrin have been shown to inhibit SARS-CoV-2 through blocking viral attachment to the host heparan sulfate proteoglycans (HSPGs) [25,26]. Small molecules that inhibit the biosynthesis of HSPGs or heparan sulfate mimetics have been shown to inhibit SARS-CoV-2 replication [27]. Therefore, we studied the impact of 4 F on perlecan, a major HSPG found within all basement membranes and cell surfaces [28]. Flow cytometry experiments showed that 4 F did not impact *in vitro* levels of perlecan compared to vehicle control in both Vero cells positive for SARS-CoV-2 spike pseudotyped lentivirus RFP expression (Figure 2c, d) and uninfected Calu3 cells (Figure 2e, f). Thus, our data suggest that the impact of 4 F on SARS-CoV-2 entry is not mediated through the HSPGs.

### Impact of 4 F on the viral entry mediator CD147

**Figure 3.**
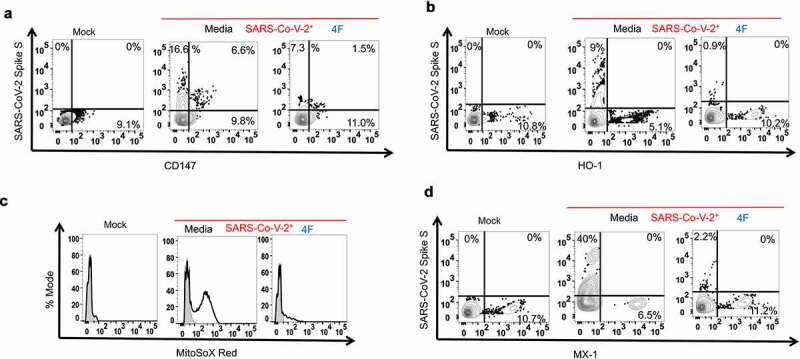
Impact of 4 F on cellular mediators involved in SARS-CoV-2 replication in lung epithelial cells. Calu3 cells were uninfected (mock) or infected with SARS-CoV-2 (MOI 0.1) and were treated with media alone (vehicle), or 4 F (10 μM) as in methods. Confluent cells were fixed 48 h post-infection (hpi) followed by processing for staining (flow cytometry) for mediators of viral entry (CD147), oxidative stress [mitochondrial reactive oxygen species (mito-ROS), Heme oxygenase 1 (HO-1)] and antiviral responses ([HO-1, myxovirus resistance MX Dynamin Like GTPase 1 (MX1)]. Flow cytometry was used in Calu3 cells to determine the median fluorescence intensity (MFI) of each target compared to a negative cell population (fluorescence minus one negative control for staining shown in light gray) (∆MFI). Representative data from three independent experiments are shown A. Flow cytometric staining for CD147 at 48 hours post-infection. B. Flow cytometric staining for the fluorochrome MitoSOX Red as a measure of mitochondrial reactive oxygen species (mito-ROS) content in Calu3 cells at 48 hours post-infection. C. Flow cytometric staining at 48 hours post-infection for HO-1. D. Flow cytometric staining at 48 hours post-infection for MX1

Prior evidence that apoA-I mimetics do not penetrate inside the cells, our data that 4 F induced a more prominent reduction in the percent of infected cells compared to the Spike S MFI in infected Vero E6 and Calu3 cells (Figure 1g-j) and that 4 F reduces entry of SARS-CoV-2 Spike S protein in epithelial cells (Figure 2) suggest that 4 F induce membrane associated antiviral effects. ACE2, MX1, and BSG/CD147 expression constitute a molecular signature that reliably differentiates COVID-19 and non-COVID-19 patients [29]. CD147 or Basigin (BSG) has been identified as potential additional host receptor for viral entry of SARS-CoV-2 [30]. CD147, as a transmembrane protein, interacts with several other proteins, such as caveolin-1 [31]. Given that 4 F depletes cellular caveolin [23], we hypothesized that 4 F attenuates CD147 protein levels in SARS-CoV-2 infected cells. Flow cytometry experiments demonstrated that CD147 protein levels were increased in SARS-CoV-2 infected compared to uninfected Calu3 cells and 4 F reduced CD147 protein expression in 4 F treated compared to vehicle treated infected Calu3 cells (Figure 3a). The impact of 4 F on CD147 in epithelial cells was the direct effect of 4 F per se and not an indirect effect related to SARS-CoV-2 replication since remdesivir did not impact levels of CD147 in SARS-CoV-2 infected cells compared to cells treated with vehicle control (Supplemental Figure 1). Thus, by reducing the viral entry host protein CD147, 4 F may reduce SARS-CoV-2 replication in lung epithelial cells.

### 4 F has antioxidant effects in SARS-CoV-2 infected epithelial cells that may contribute to antiviral cellular responses

We next determined potential cellular mediators that may mediate the antiviral effect of 4 F in SARS-CoV-2 infected epithelial cells. Previous *in vitro* studies and *in vivo* data in mice have shown that 4 F increases the expression and activity of two antioxidant enzymes, heme oxygenase 1 (HO-1) and superoxide dismutase (SOD) in endothelial [32,33] and epithelial malignant cells [16]. Because these enzymes modulate cellular oxidative stress that is an instigator of viral infections [34] and given that induction of HO-1 may be a potent antiviral strategy for several viruses including SARS-CoV-2 [35], we hypothesized that 4 F upregulates HO-1 in epithelial cells. Flow cytometry experiments demonstrated that HO-1 protein levels were decreased in SARS-CoV-2 infected compared to uninfected Calu3 cells and 4 F increased HO-1 protein expression in 4 F treated SARS-CoV-2 compared to vehicle treated infected Calu3 cells (Figure 3b). Consistent with the antioxidant effect of 4 F, the MFI of MitoSOX red, a measure of cellular content for mitochondrial reactive oxygen species (mito-ROS) was also reduced in 4 F treated compared to vehicle treated infected Calu3 cells (Figure 3c). The impact of 4 F on HO-1 and mito-ROS in epithelial cells was the direct effect of 4 F per se and not an indirect effect related to SARS-CoV-2 replication since remdesivir did not impact levels of mito-ROS and HO-1 in SARS-CoV-2 infected cells compared to cells treated with vehicle control (Supplemental Figure 1). Thus, given established role of reduced levels of HO-1^35^ and increased mito-ROS in replication of viruses [34], the antioxidant effects of 4 F in epithelial cells may contribute to its effects against SARS-CoV-2.

### Impact of 4 F on interferon antiviral responses in SARS-CoV-2 infected epithelial cells

Heme oxygenase 1 (HO-1) is an inducer of the myxovirus resistance (MX) genes [36] that encode GTPases that are part of the antiviral response induced by type I/III IFNs [37]. MX1 is a key antiviral effector in COVID-19 patients [29]. We therefore determined the impact of 4 F on protein levels of MX1 in infected epithelial cells. Flow cytometry experiments demonstrated that MX1 protein levels were decreased in SARS-CoV-2 infected compared to uninfected Calu3 cells and 4 F increased MX1 protein expression in 4 F- compared to vehicle treated infected Calu3 cells (Figure 3d). The impact of 4 F on MX1 in epithelial cells was the direct effect of 4 F per se and not an indirect effect related to SARS-CoV-2 replication since remdesivir did not impact levels of MX1 in SARS-CoV-2 infected cells compared to cells treated with vehicle control (Supplemental Figure 1). These data suggest that the antiviral activity of 4 F against SARS-CoV-2 is partially mediated through the interferon pathway.

### 4 F attenuates apoptosis associated with SARS-CoV-2 infection in epithelial cells

**Figure 4.**
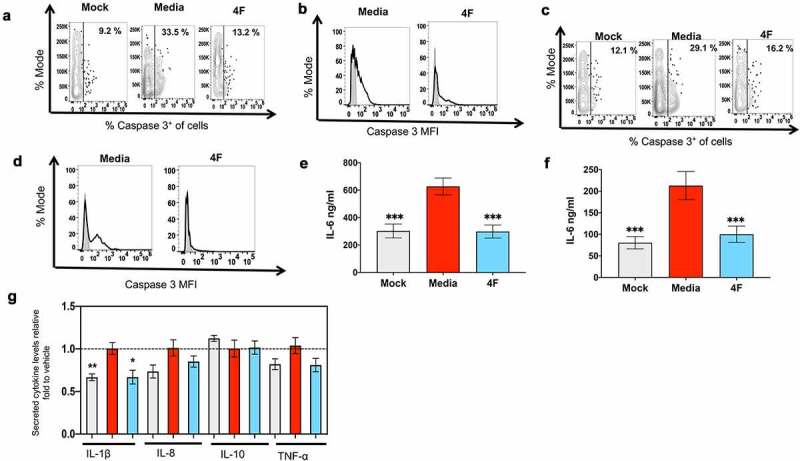
4 F restricts apoptosis and inflammatory responses associated with SARS-CoV-2 infection in lung epithelial cells. A-D. Vero E6 and Calu3 cells were uninfected (mock) or infected with SARS-CoV-2 (MOI 0.1) and were treated with media alone (vehicle), or 4 F (10 μM) as in methods. Confluent cells were fixed 48 h post-infection (hpi) followed by processing for flow cytometry. **A, B**. Flow cytometry was used in Vero E6 cells to determine the percent of viable cells positive for cleaved caspase 3 (a) and the median fluorescence intensity (MFI) of cleaved caspase 3 (b) compared to a negative cell population (shown in light gray). Representative data from three independent experiments are shown. **C, D**. Flow cytometry was used in Calu3 cells to determine the percent of viable cells positive for cleaved caspase 3 (c) and the MFI of cleaved caspase 3 (d) compared to a negative cell population. Representative data from three independent experiments are shown. **E, F**. ELISA was used to determine protein levels of IL-6 (ng/ml) secreted by Calu3 (e) and Vero-E6 (f) cells 48 hours post SARS-CoV-2 infection. The compared groups were uninfected cells (mock, light gray), cells infected with SARS-CoV-2 (SARS-CoV-2^+^, red), and cells infected with SARS-CoV-2 treated with 4 F (light blue). Data represent the mean ± standard error of means (SEM), representing three independent experiments conducted at least in triplicate. The Mann-Whitney was used to compare each group relative to the vehicle control and the p value for this comparison is shown above each column (***p < 0.001). **G**. Luminex immunoassays were used to determine protein levels of IL-1β, IL-8, IL-10, TNF-α (pg/ml) secreted by Calu-3 cells 48 hours post SARS-CoV-2 infection. The mean value of each measurement (protein levels in cell culture supernatants in pg/ml) was normalized by the mean value of each measurement in the vehicle group and expressed as fold to the mean of the vehicle control group. Data represent the mean ± SEM, representing three independent experiments conducted at least in triplicate. The Kruskal-Wallis statistical test was used to compare 3 groups and the Mann-Whitney was used to compare each group relative to the vehicle control. The p value for this comparison is shown above each column (*p < 0.05, **p < 0.01, ***p < 0.001)

Next, we assessed the impact of 4 F on cellular apoptosis associated with SARS-CoV-2 infection, given that increased apoptosis of epithelial cells and pneumocytes is associated with lung injury and severe COVID-19 [38] and prior evidence that 4 F attenuates viral induced cellular apoptosis [17]. Flow cytometry showed that 4 F attenuated SARS-CoV-2-induced increase in both the percent of infected cells that were positive for cleaved caspase 3 (Figure 4a, c) and the MFI of cleaved caspase 3 per cell (Figure 4b, d) in both Vero E6 (Figure 4a, b) and Calu3 cells (Figure 4c, d). Thus, our results show that, 4 F not only has antiviral activity against SARS-CoV-2 but also has anti-apoptotic effects associated with SARS-CoV-2 infection in epithelial cells that may alleviate severe lung injury in COVID-19.

### 4 F attenuates release of proinflammatory cytokines by SARS-CoV-2-infected cells

Next, we assessed the anti-inflammatory impact of 4 F on SARS-CoV-2 infected cells, given prior data that 4 F attenuated release of IL-6 by influenza-infected epithelial cells [17] and that IL-6 is associated with lung injury and severe COVID-19 [38]. Using ELISA and Luminex immunoassays, we found that 4 F attenuated SARS-CoV-2-induced increase in secretion of IL-6 by both Calu3 (Figure 4e) and Vero E6 (Figure 4f) cells and secretion of IL-1β in cell culture supernatants of SARS-CoV-2 infected Calu3 cells (Figure 4g). There was no impact of SARS-CoV-2 or 4 F on IL-8, IL-10 and TNF-α secreted by Calu3 cells (Figure 4g). The impact of 4 F on IL-6 in epithelial cells was the direct effect of 4 F per se and not an indirect effect related to SARS-CoV-2 replication since remdesivir did not impact levels of IL-6 in SARS-CoV-2 infected cells compared to cells treated with vehicle control (Supplemental Figure 1). Thus, 4 F attenuated secretion of both IL-6 and IL-1β by infected SARS-CoV-2 cells.

## Discussion

Our data that 4 F is antiviral against SARS-CoV-2 are consistent with prior evidence that apoA-I mimetic peptides have antiviral activity [7]. The main therapeutic avenues to halt respiratory virus infection are targeting the virus directly or the host system. Although the first strategy is highly efficient, it is limited by the resistance of the virus strain [39,40]. Although 4 F had less antiviral efficacy than remdesivir, most likely related to its mechanism of action through cellular pathways rather than inhibition of the virus per se, by targeting host antiviral mechanisms 4 F may have antiviral activity against SARS-CoV-2 variants such as the United Kingdom (UK), B.1.1.7 variant. In addition, its potent pleiotropic anti-inflammatory activity makes it an attractive therapeutic option against COVID-19, especially since its safety has been tested in clinical trials in humans in both oral and systemic formulations [13,14].

ApoA-I mimetic peptides reduce not only high levels of cholesterol but also bioactive lipids that are increased in comorbidities like cardiovascular disease. Targeting aberrant signaling of bioactive lipids can be a therapeutic strategy for COVID-19 [41,42]. We have shown that apoA-I mimetics bind [43] and remove [44] oxidized lipids from the circulation, thus reducing the oxidative stress that contributes to endothelial dysfunction, pulmonary hypertension and lung cancer in murine models of lung disease [11,45–47]. Bioactive lipids may also disrupt epithelial cell tight junctions either directly [48] or through lipid raft disruption [49,50] or TNF-α signaling [51]. Inhibition of proinflammatory oxidized phospholipids mediated the *in vitro* antiviral activity of 4 F against influenza infection in type II pneumocytes [17]. Thus, by depleting elevated intestinal and systemic levels of bioactive lipids (in the setting of comorbidities), 4 F may alter cellular signaling in remote epithelial tissues and upregulate antioxidant enzymes (like HO-1 and SOD) in epithelial and endothelial cells [16,32,33]. Consistent with our data, therapies with anti-viral properties that raise HO-1 such as statins, could be used for treatment of COVID-19 [35,52]. Collectively, these antioxidant effects of 4 F (reduction in bioactive lipids, upregulation of HO-1) may contribute to inhibition of SARS-CoV-2 replication in epithelial cells.

Consistent with the established antioxidant effects of 4 F, we found that 4 F decreased the cellular content of SARS-CoV-2 infected lung epithelial cells for mito-ROS. Increased mito-ROS may contribute to increased SARS-CoV-2 replication through multiple mechanisms. Mito-ROS interact with coatomer coat protein complex, which is a proviral host factor in SARS-CoV replication [53]. Mito-ROS are key inducers of the mitochondrial permeability transition pore that promotes coronavirus replication [54]. Mito-ROS trigger MEK [55], MNK1 [56] and MAPK signaling pathways [57–59] that propagate viral protein synthesis and SARS-Co-V replication [60–62]. Mito-ROS also induce aberrant ER stress, lipid peroxidation, alterations of membranes and proteins and activation of cytosolic phospholipases [63–65]; collectively these changes may lead to increased viral replication [66]. Thus, by targeting mito-ROS and oxidized lipids that induce multiple cellular signaling pathways that are essential for viral replication in epithelial cells, 4 F may target SARS-CoV-2 replication.

Increased cellular mito-ROS also downregulate induction of interferon during viral infections [67]. We found that the antiviral activity of 4 F was more potent in interferon competent Calu3 cells compared to interferon deficient monkey epithelial cells. 4 F also increased HO-1, an inducer of IFN-I responses during SARS-CoV-2 infection [36]. Our *in vitro* data are consistent with *in vivo* animal studies that oral apoA-I mimetic peptides upregulate *in vivo* IFNβ1 and MX1 levels in the lungs of mice with lung cancer [11]. Collectively, this evidence supports that the antiviral activity of 4 F against SARS-CoV-2 is partially mediated through the interferon pathway.

It is imperative to develop therapeutic agents that inhibit viral replication *and* viral induced “cytokine storm”, severe lung injury and lethality [68,69]. We found that 4 F has anti-inflammatory activity in the setting of SARS-CoV-2 infection of epithelial cells. Our data is consistent with prior evidence that 4 F has pleiotropic anti-inflammatory effects and reduces the expression of NF‐κB pathway, IL-6 and IL-1β secretion and ultimately lung injury during infections [17,70]. Heme oxygenase-1 (HO-1) can also abrogate leukocyte recruitment and tissue injury after endotoxin (LPS) stimulation *in vivo* [71]. Oral apoAI mimetic peptides like 6 F (Tg6F) directly bind not only bioactive lipids [8,72,73] but also microbial products like LPS to alter intestinal immune cells and the expression of genes including IFNβ1 and MX1 levels, leading to similar changes in lung and reduced lung disease such as cancer [11]. Thus, by attenuating altered gut microbiome and increased microbial products (such as LPS) in patients with comorbidities like cardiovascular disease [74,75], 4 F reduces activation of the TLR and NF-κB pathways and associated lung injury in infections like SARS-CoV-2.

Given that mechanistic *in vitro* studies suggest that membrane association is required for the pro-apoptotic activity of SARS-CoV-2 ORF3a [76] and the known impact of 4 F on cellular membranes through reduction in lipid content and lipid rafts, the anti-apoptotic effects of 4 F on SARS-CoV-2 infected cells may be related to its impact on cellular membranes. ApoA-I also protects mitochondria by multiple mechanisms [77] that may be related to SARS-CoV-2 induced mitochondrial injury and cellular apoptosis. Given that 4 F depletes cellular caveolin [23], a component of lipid raft which may be specialized for delivering lipids to multiple cellular compartments in airway epithelial cells [78], 4 F may have several favorable effects on membrane associated mitochondrial proteins that are involved in apoptosis.

Our study has limitations. The results presented here are in an isolated *in vitro* system, devoid of any immune system cellular components that are thought to be critical to the many aspects of the COVID-19 viral response *in vivo*. However, our prior studies in mice, may explain how oral 4 F that has low systemic absorption [9,11,12], by targeting gut inflammation, altered microbiome products, lipids and bioactive lipids, can have a major favorable impact on systemic oxidative stress, inflammation and lung disease and can upregulate *in vivo* pathways (such as HO-1) [16,32,33] that are key for host antiviral and anti-inflammatory responses against SARS-CoV-2 infection. ApoA-I mimetic peptides have pleotropic cellular effects and it is possible that other mechanisms of action may explain their antiviral activity against SARS-CoV-2. For example, 4 F depletes cholesterol from cellular membranes and caveolin [23], a component of lipid rafts which plays essential roles in viral entry and fusion [79]. Depletion of cholesterol from cellular membranes also inhibits SARS-CoV-2 infection [79]. Thus, by depleting elevated levels of lipids (in the setting of comorbidities like dyslipidemia and cardiovascular disease) from cellular membranes, 4 F may inhibit SARS-CoV-2 replication in epithelial cells (respiratory or intestinal).

Importantly, both statins [80] and 4 F [23] reduce abundance of lipid rafts, the “point of entry” for several viruses, by depleting them of lipids responsible for their stability [81]. Both animal [82] and human [83] studies have shown that 4 F and statins may have major additive *in vivo* effects on altered lipid levels [82–84]. Thus, further studies are needed to assess the potential antiviral mechanisms of action of 4 F through cellular lipids and potential additive antiviral effects with statins.

In conclusion, our current *in vitro* data suggest that 4 F has not only antiviral and antiapoptotic but also antioxidant and anti-inflammatory effects on SARS-CoV-2 infected epithelial cells, suggesting that it may have multiple favorable therapeutic effects in COVID-19. This strategy needs to be validated in further animal and human studies and can be particularly useful for COVID-19 for several reasons: 1) can be used as long-term preexposure prophylaxis against viral infections in high-risk groups that have comorbidities and are either immunocompromised or have contraindications for a vaccine; 2) can have favorable impact on pathogenesis of chronic COVID syndrome (CCS) where inflammatory mechanisms are implicated [85,86]; 3) can attenuate potentially long-term cardiovascular effects associated with COVID-19 [87]; 4) there is no single oral safe potent antiviral for COVID-19, to date. Clinical trials are needed to investigate the role of 4 F in prophylaxis and treatment of COVID-19 disease across different levels of severity.

## Supplementary Material

Supplemental Material

## Data Availability

Requests for data should be sent to the corresponding author.
